# Real-world evidence on the dosing and safety of C.E.R.A. in pediatric dialysis patients: findings from the International Pediatric Dialysis Network registries

**DOI:** 10.1007/s00467-023-05977-z

**Published:** 2023-08-11

**Authors:** Laura Kohlhas, Milena Studer, Loes Rutten-Jacobs, Sylvie Meyer Reigner, Anja Sander, Hui-Kim Yap, Karel Vondrak, Paula A. Coccia, Francisco Cano, Claus Peter Schmitt, Bradley A. Warady, Franz Schaefer, IPDN collaborators, Karel Vondrak, Paula A. Coccia, Yok-Chin Yap, Hui-Kim Yap, Francisco Cano, Il Soo Ha, Rainer Büscher, Lars Pape, Charlotte Samaille, Bradley A. Warady, Dorota Drozdz, Koen van Hoeck, Juan Jose Vanegas, Pedro Zambrano, Marcus Weitz, Maria Szczepanska

**Affiliations:** 1 Institute of Medical Biometry, University of Heidelberg, Heidelberg, Germany; 2 F. Hoffmann-La Roche Ltd, Basel, Switzerland; 3 Department of Paediatrics, Yong Loo Lin School of Medicine, National University of Singapore, Singapore, Republic of Singapore; 4 Department of Pediatrics and Transplantation Center, University Hospital Motol, 2nd Medical Faculty Prague, Charles University Prague, Prague, Czech Republic; 5 Division of Pediatric Nephrology, Hospital Italiano de Buenos Aires, Buenos Aires, Argentina; 6 Division of Pediatric Nephrology, Hospital Dr. Luis Calvo Mackenna, Facultad de Medicina, Universidad de Chile, Santiago, Chile; 7 Division of Pediatric Nephrology, Center for Pediatrics and Adolescent Medicine, Heidelberg, Germany; 8 Division of Pediatric Nephrology, Children’s Mercy Kansas City, Kansas City, MO USA

**Keywords:** Chronic kidney disease, Continuous erythropoietin receptor activator, Real-world study, Peritoneal dialysis, Hemodialysis

## Abstract

**Background:**

This retrospective real-world study used data from two registries, International Pediatric Peritoneal Dialysis Network (IPPN) and International Pediatric Hemodialysis Network (IPHN), to characterize the efficacy and safety of continuous erythropoietin receptor activator (C.E.R.A.) in pediatric patients with chronic kidney disease (CKD) on peritoneal dialysis (PD) or hemodialysis (HD).

**Methods:**

IPPN and IPHN collect prospective data (baseline and every 6 months) from pediatric PD and HD centers worldwide. Demographics, clinical characteristics, dialysis information, treatment, laboratory parameters, number and causes of hospitalization events, and deaths were extracted for patients on C.E.R.A. treatment (IPPN: 2007–2021; IPHN: 2013–2021).

**Results:**

We analyzed 177 patients on PD (median age 10.6 years) and 52 patients on HD (median age 14.1 years) who had ≥ 1 observation while being treated with C.E.R.A. The median (interquartile range [IQR]) observation time under C.E.R.A. exposure was 6 (0–12.5) and 12 (0–18) months, respectively. Hemoglobin concentrations were stable over time; respective means (standard deviation) at last observation were 10.9 (1.7) g/dL and 10.4 (1.7) g/dL. Respective median (IQR) monthly C.E.R.A. doses at last observation were 3.5 (2.3–5.1) µg/kg, or 95 (62–145) µg/m^2^ and 2.1 (1.2–3.4) µg/kg, or 63 (40–98) µg/m^2^. Non-elective hospitalizations occurred in 102 (58%) PD and 32 (62%) HD patients. Seven deaths occurred (19.8 deaths per 1000 observation years).

**Conclusions:**

C.E.R.A. was associated with efficient maintenance of hemoglobin concentrations in pediatric patients with CKD on dialysis, and appeared to have a favorable safety profile. The current analysis revealed no safety signals.

**Graphical abstract:**

**Figure:**
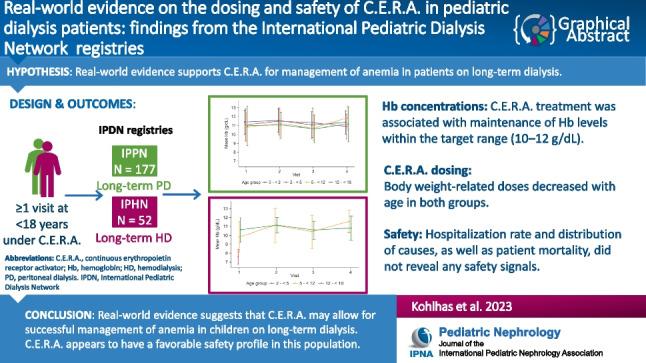
A higher resolution version of the Graphical abstract is available as Supplementary information

**Supplementary Information:**

The online version contains supplementary material available at 10.1007/s00467-023-05977-z.

## Introduction

Anemia due to chronic kidney disease (CKD) is characterized by inadequate levels of erythropoietin (EPO) and leads to cardiovascular dysfunction [1, 2], reduced physical activity [3, 4], and impairs quality of life [5, 6], and is associated with increased hospitalization and mortality rates in both adults and children [7–9]. The currently available treatment options include short-acting erythropoiesis-stimulating agents (ESAs) such as recombinant human erythropoietin (epoetin alfa and beta), and longer-acting ESAs such as hyperglycosylated (darbepoetin alfa) and pegylated (continuous erythropoietin receptor activator [C.E.R.A.]) recombinant human erythropoietin, which require less frequent dosing due to their prolonged half-life. The 4-weekly administration schedule of C.E.R.A. appears highly attractive for the pediatric population as it reduces the frequency of injections and allows in-center management of anemia, even in patients on home peritoneal dialysis (PD).

In 2018, the US Food and Drug Administration (FDA) granted approval for C.E.R.A. to be administered intravenously in pediatric patients on hemodialysis (HD) aged 5 to 17 years who are converting from another ESA, based on the successful completion of a clinical trial (DOLPHIN) [10]. Another trial (SKIPPER) performed in pediatric patients, including children < 5 years old, on HD, PD, or not on dialysis, utilizing subcutaneous administration of C.E.R.A., was completed recently [11, 12]. The data from these two pediatric studies showed that administration of C.E.RA. every 4 weeks was able to maintain hemoglobin (Hb) concentration levels within the targeted range (10–12 g/dL) when administered to patients with CKD on dialysis or not on dialysis, when switching from stable maintenance treatment with epoetin alfa, epoetin beta, or darbepoetin alfa, using a defined conversion factor [10–12]. To supplement the database from both pediatric clinical trials, additional aggregated safety data and patient-level data on C.E.R.A. dosing and Hb values from the International Pediatric Dialysis Network (IPDN) was analyzed to provide data from real-world clinical practice.

The IPDN entertains two registries: the International Pediatric Peritoneal Dialysis Network (IPPN) registry for children on long-term PD, and the International Pediatric Hemodialysis Network (IPHN) registry for children on HD. We analyzed C.E.R.A. dosing, resultant Hb concentrations, and safety data (number and causes of hospitalizations and deaths) in pediatric patients on HD or PD who were followed in these registries while being treated with C.E.R.A. in a real-world setting.

## Methods

In this retrospective observational study, data on patients who received C.E.R.A. were extracted from two existing registries within the IPDN, i.e*.* the IPPN registry and the IPHN registry. Approval for the IPDN registries protocol was obtained from independent ethics committees and institutional review boards as required by each participating center. Written parental consent and, whenever appropriate, assent from patients was obtained.

The registries collect prospective (baseline and every 6 months) information from 137 pediatric PD and HD centers in 44 countries around the globe [13]. At baseline (first observation), patients might have already received 1–5 monthly doses of C.E.R.A. The participating centers are asked to enroll all incident and prevalent patients and enter data longitudinally until long-term PD or HD is discontinued. Data input into the registries is performed exclusively via an internet-based web platform (www.pedpd.org). Data entries are automatically checked for plausibility and completeness. Data protection is ensured by pseudonymized data input.

Inclusion criteria of the population analyzed in this study were receipt of long-term PD or HD with at least one observation in the registry database while being treated with C.E.R.A., and age < 18 years at the first observation while on C.E.R.A. The data collection periods were from Q2/2007 through Q2/2021 for IPPN and from Q1/2011 through Q2 2021 for IPHN.

The primary variables of interest were the relationship between C.E.R.A. dosing and Hb concentrations, as well as safety variables (i.e. death and hospitalizations [number and causes]). Since the registries collect data every 6 months, treatment information is available as 6-month snapshots at each observation.

The primary objectives were addressed by conducting descriptive analyses of C.E.R.A. dose, Hb concentration, and safety data, as well as selected patient characteristics. Between-group differences were evaluated for significance by Student’s t-test. Pearson correlation coefficients were calculated to express associations between variables. Statistical significance was defined as *p* < 0.05.

Absolute and relative frequencies were calculated for hospitalizations and leading causes of hospitalizations. For continuous variables, mean and standard deviation (SD), or median and first and third quartile (interquartile range [IQR]) are given depending on the distribution of the variable.

In cases where characteristics are given for the first and last observation, patients with only one observation in the registry while on C.E.R.A. were included in both groups. After study completion, additional analyses of Hb concentrations and C.E.R.A. dose were carried out for a subgroup of patients with two or more observations in the registry. Only the first period of C.E.R.A. use was considered in the analysis; observations where C.E.R.A. was restarted after a treatment break were not considered.

## Results

**Fig. 1:**
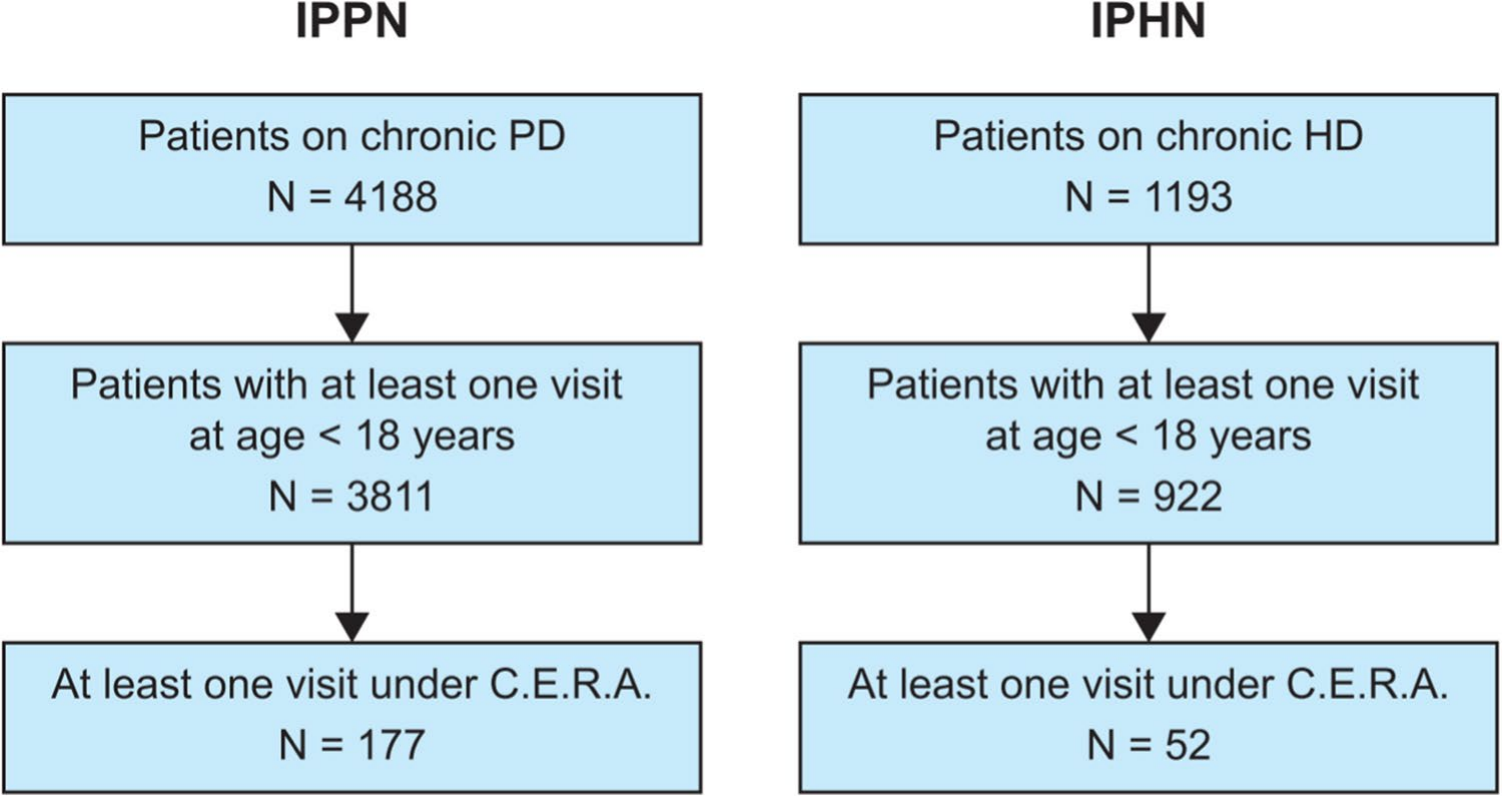
Study CONSORT diagram. *HD*, hemodialysis; *IPHN*, International Pediatric Hemodialysis Network; *IPPN*, International Pediatric Peritoneal Dialysis Network; *PD*, peritoneal dialysis; *C.E.R.A.*, continuous erythropoietin receptor activator

A total of 229 pediatric patients on dialysis (177 PD, 52 HD) receiving C.E.R.A. were analyzed in this study. The selection process is depicted in Fig. 1. Among all patients aged < 18 years followed in the registries, 5% of patients in the IPPN and 6% in the IPHN registry received C.E.R.A. with one or more observations while on C.E.R.A. captured in the registries. The patients were treated in 18 pediatric dialysis centers in 11 countries (Table 1).

**Table 1:** Patient characteristics

	PD	HD
	*n* = 177	*n* = 52
Female sex, n (%)	67 (38%)	20 (39%)
Mean age, years (± SD)	9.6 (5.5)	13.0 (3.8)
Age 0 to < 2 years, n (%)	19 (11%)	-
Age 2 to < 5 years, n (%)	31 (18%)	2 (4%)
Age 5 to < 12 years, n (%)	55 (31%)	17 (33%)
Age 12 to < 18 years, n (%)	72 (41%)	33 (63%)
Geographic region, n (%)		
Europe*	94 (53%)	50 (96%)
Asia†	47 (27%)	1 (2%)
Latin America‡	36 (20%)	0 (0.0%)
North America§	0 (0%)	1 (2%)
Primary renal diagnosis, n (%)		
- CAKUT	92 (52%)	34 (65%)
- Glomerular disorders	70 (38%)	17 (33%)
- Other	15 (9%)	1 (2%)
Defined syndrome¶, n (%)	31 (18%)	9 (17%)
Mean body weight, kg (± SD)	28.4 (16.5)	42.3 (19.5)
Mean body surface area, m^2^ (± SD)	1.0 (0.4)	1.3 (0.4)
Mean systolic blood pressure, mmHg (± SD)	113.9 (18.7)	128.5 (15.3)
Mean diastolic blood pressure, mmHg (± SD)	71.2 (15.2)	76.6 (12.7)
Median time on dialysis, months (IQR)	11.0 (3.1–27.5)	2.6 (1.5–9.3)
C.E.R.A. route of administration, n (%)		
- Intravenous	46 (38%)	49 (94%)
- Subcutaneous	75 (62%)	3 (6%)
- Missing	56	
Prior ESA, n (%)		
- Alfa erythropoietin	26 (23.3%)	2 (15.4%)
- Beta erythropoietin	55 (49.1%)	3 (23.1%)
- Darbepoetin	16 (14.3%)	7 (53.8%)
- Delta erythropoietin	11 (9.8%)	0 (0.0%)
- No	4 (3.6%)	1 (7.7%)
- Missing	65	39
ACE inhibitor/ARB, n (%)		
- No	100 (59.9%)	38 (73.1%)
- Yes	67 (40.1%)	14 (26.9%)
- Missing	10	
Iron therapy, n (%)		
- No	38 (21.5%)	8 (15.4%)
- Yes	139 (78.5%)	44 (84.6%)
Median serum ferritin, ng/ml (IQR)	183 (92–333)	265 (148–367)
Median transferrin saturation, % (IQR)	33.7 (21.6–38.7)	27.9 (20.0–36.0)

Data are given as n (%), mean (SD), or median (interquartile range) as appropriate

*ACE* angiotensin-converting-enzyme, *ARB* angiotensin II receptor blocker, *CAKUT* congenital anomalies of the kidney and urinary tract, *C.E.R.A.* continuous erythropoietin receptor activator, *ESA* erythropoiesis-stimulating agent, *HD* hemodialysis, *IQR* interquartile range, *PD* peritoneal dialysis, *SD* standard deviation

*Belgium, Czech Republic, France, and Germany

†South Korea, Malaysia, and Singapore

‡Argentina, Chile, and Colombia

§The United States

¶Defined syndromes are disorders that also affect organs other than the kidney, such as Bardet-Biedl syndrome, VACTERL syndrome, or even Down syndrome. Patients with defined syndromes often have additional comorbidities that may modify outcomes. They are less relevant with respect to anemia treatment

The median age at first observation on C.E.R.A. was 10.6 (IQR 4.2–14.6) and 14.1 (10.4–16.2) years for the PD and HD cohorts, respectively. A summary of the characteristics of the patients on PD or HD at their first observation under C.E.R.A. is presented in Table 1. The median observation time under C.E.R.A. exposure was 6.1 (0–12.5) months and 11.9 (0–17.9) months for patients on PD and HD, respectively (Table 2). An additional analysis was completed for a subgroup of patients with two or more observations in the registry: Supplementary Table 1 shows the characteristics of these patients and Supplementary Table 2 shows the C.E.R.A. duration of exposure.

**Table 2:** C.E.RA. duration of exposure

	PD	HD
	*n* = 177	*n* = 52
Median duration of exposure, months (IQR)	6.1 (0.0–12.5)	11.9 (0.0–17.9)
C.E.R.A. duration of exposure, n (%)		
- ≤ 6 months	86 (48.6)	20 (38.5)
- 6–12 months	38 (21.5)	6 (11.5)
- 12–18 months	22 (12.4)	13 (25.0)
- 18–24 months	12 (6.8)	3 (5.8)
- 24–36 months	9 (5.1)	8 (15.4)
- 36–48 months	5 (2.8)	1 (1.9)
- 48–60 months	2 (1.1)	1 (1.9)
- 60–71 months	2 (1.1)	0 (0.0)
- 84–96 months	1 (0.6)	0 (0.0)

*C.E.R.A.* continuous erythropoietin receptor activator, *HD* hemodialysis, *IQR* interquartile range, *PD* peritoneal dialysis

### Hb concentrations

**Fig. 2:**
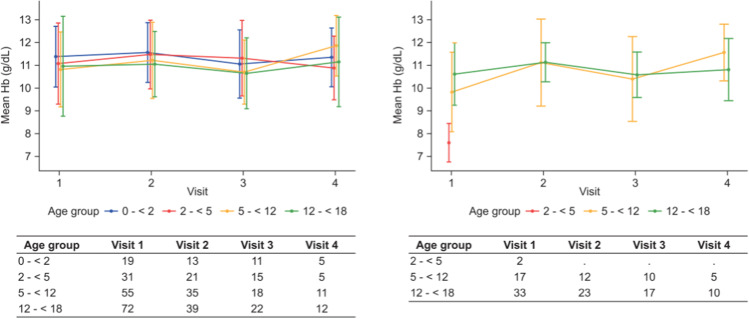
Hemoglobin concentrations over time per age group for PD (left panel) and HD cohort (right panel). Data are shown as mean ± SD per 6-monthly observation (visit). *HD*, hemodialysis; *PD*, peritoneal dialysis; SD, standard deviation

The mean (SD) Hb concentration was 11.0 (1.9) g/dL at first observation and 10.9 (1.7) g/dL at last observation under C.E.R.A. in the PD cohort, and 10.2 (1.6) g/dL at first and 10.4 (1.7) g/dL at last observation under C.E.R.A. in the HD cohort (Table 3). In both cohorts, Hb concentrations were stable over time; Hb concentrations per age group at the first observation (177 PD, 52 HD), second observation (108 PD, 35 HD), third observation (66 PD, 27 HD), and fourth observation (33 PD, 15 HD) are shown in Fig. 2. At last observation, 47% of the children on PD and 48% of those on HD had a Hb value in the target range, 10–12 g/dL [14]; Hb values above the target range were seen in 25% and 14% of patients on PD and HD, respectively, and values below the target range were seen in 28% and 39% of patients on PD and HD, respectively. When comparing results between age groups (age groups < 2 years; 2 to < 5 years, 5 to < 12 years and 12 to < 18 years), mean Hb concentrations were stable over time and within the target range for all age groups in the PD cohort (Fig. 2). Hb concentrations were generally lower in the HD cohort. However, there were only 2 patients in the age group 2 to < 5 in the HD cohort and there were only data from one observation available. Similar Hb values were seen in patients with two or more observations in the registry (Supplementary Table 3): the mean (SD) Hb concentration was 11.1 (1.9) g/dL at first observation and 11.0 (1.6) g/dL at last observation under C.E.R.A. in the PD cohort (108 patients; all routes of administration of PD [subcutaneous and intravenous]), and 10.4 (1.4) g/dL at first and 10.7 (1.7) g/dL at last observation under C.E.R.A. in the HD cohort (35 patients).

**Table 3:** Hemoglobin level and C.E.R.A. dose (absolute and normalized to body weight or body surface area) at first and last observation on treatment, stratified by route of administration

	PD subcutaneous		PD intravenous		PD all routes		HD	
	First obs *n* = 75	Last obs *n* = 73	First obs *n* = 46	Last obs *n* = 53	First obs *n* = 177	Last obs *n* = 177	First obs *n* = 52	Last obs *n* = 52
Hemoglobin, g/dL	11.2 (2.0)	10.9 (1.8)	10.8 (1.8)	11.2 (1.4)	11.0 (1.9)	10.9 (1.7)	10.2 (1.6)	10.4 (1.7)
C.E.R.A. monthly dose, µg	100 (50–161)	100 (50–161)	100 (50–120)	100 (50–120)	100 (50–120)	100 (50–150)	107 (80–129)	80 (54–129)
C.E.R.A. monthly dose, µg/kg	3.5 (2.6–5.4)	3.6 (2.5–5.1)	3.5 (2.3–5.3)	3.0 (1.9–4.7)	3.4 (2.3–5.4)	3.5 (2.3–5.1)	2.9 (1.7–4.0)	2.1 (1.2–3.4)
C.E.R.A. monthly dose, µg/m^2^	96 (73–131)	101 (69–141)	95 (60–139)	89 (54–118)	94 (67–144)	95 (62–145)	89 (59–115)	63 (40–98)

Hemoglobin is given as mean (SD), doses as median (interquartile range). The results of three patients on HD treated subcutaneously are not shown

*C.E.R.A.* continuous erythropoietin receptor activator, *HD* hemodialysis, *obs* observations, *PD* peritoneal dialysis, *SD* standard deviation

### C.E.R.A. dosing

In the PD cohort, the median (IQR) C.E.R.A. monthly dose at first observation was 100 (50–120) µg, 3.4 (2.3–5.4) µg/kg body weight, or 94 (67–144) µg/m^2^ body surface area, and at last observation 100 (50–150) µg, 3.5 (2.3–5.1) µg/kg, or 95 (62–145) µg/m^2^. The median C.E.R.A. dose in the PD cohort was similar in patients who received subcutaneous and intravenous administrations. In the HD cohort, the median (IQR) monthly C.E.R.A. dose at first observation was 107 (80–129) µg, 2.9 (1.7–4.0) µg/kg, or 89 (59–115) µg/m^2^, and at last observation 80 (54–129) µg, 2.1 (1.2–3.4) µg/kg, or 63 (40–98) µg/m^2^ (Table 3). C.E.R.A. monthly doses were comparable for patients with two or more observations in the registry (Supplementary Table 3).

At the last observation, information about the route of administration was available from all patients on HD and from 126 of the 177 patients on PD. While all but 3 patients on HD received C.E.R.A. intravenously, 62% of the patients on PD with available information received subcutaneous, and 38% intravenous injections at the first observation. Six of 75 patients on PD were switched from subcutaneous to intravenous administration during the observation period. The average C.E.R.A. doses applied and Hb concentrations achieved at first and last observation by administration route are displayed in Table 3. Supplementary Table 3 shows the number of patients who received subcutaneous PD and patients who received intravenous PD with two or more observations at first and last observation, and the Hb concentrations and C.E.R.A. monthly dose.

The absolute monthly C.E.R.A. dose increased with age. In the PD cohort, the median (IQR) dose at first observation was 30 (30–100) µg in infants younger than 2 years, 75 (30–107) in children 2 to < 5 years old and 80 (60–100) µg in children 5 to < 12 years old compared with 114 (75–161) µg in the adolescent age group (12 to < 18 years). In the HD cohort, the absolute dose was 91 (54–129) µg in children 2 to < 5 years old and 86 (80–107) µg in children 5 to < 12 years old compared with 107 (80–129) µg in the adolescent age group. At the last observation, the median monthly C.E.R.A. dose in the PD cohort ranged from 50 (30–100) µg in infants younger than 2 years to 146 (78–170) µg in adolescents, and in the HD cohort from 91 (54–129) µg in two children 2 to < 5 years old to 80 (54–129) µg in the adolescents.

**Fig. 3:**
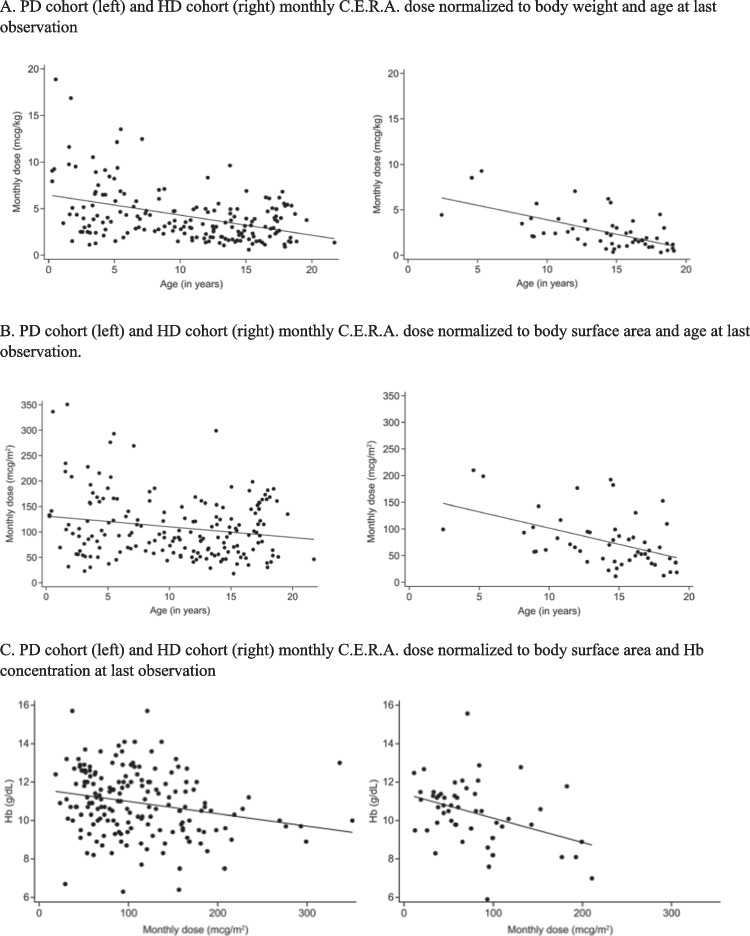

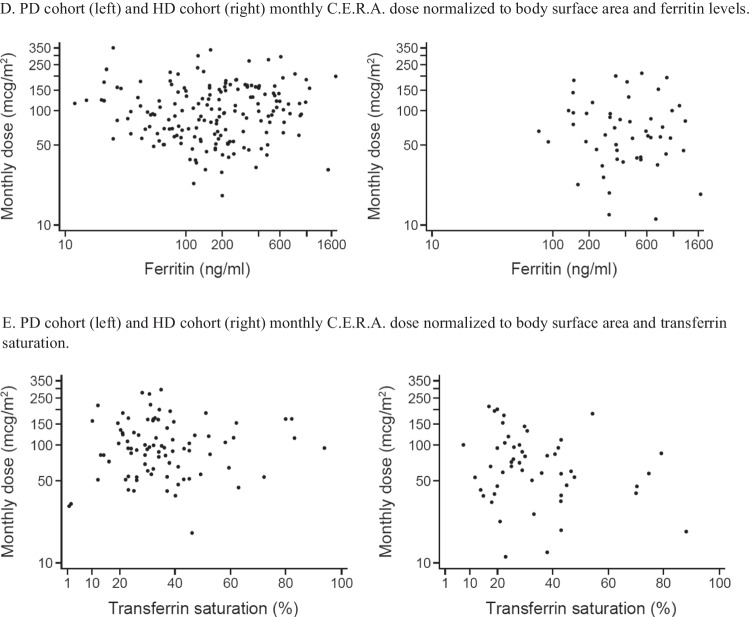
Monthly C.E.R.A. dose. C.E.R.A. dose normalized to body surface area. C.E.R.A., continuous erythropoietin receptor activator; *HD*, hemodialysis; *PD*, peritoneal dialysis

 Whereas absolute C.E.R.A. doses were positively correlated with age, weight-related doses markedly decreased with age in both cohorts (Fig. 3). In the children on PD aged < 2, 2 to < 5, 5 to < 12, and 12 to < 18 years, respectively, the median (IQR) C.E.R.A. monthly dose was 7.9 (4.9–11.6), 5.4 (3.0–7.5), 3.2 (2.7–4.9), and 2.6 (1.9–3.9) µg/kg at first observation and 5.1 (3.4–9.8), 5.2 (2.7–6.9), 3.0 (2.3–4.7), and 3.0 (1.7–4.5) µg/kg at last observation. In children on HD aged 2 to < 5, 5 to < 12, and 12 to < 18 years, the median monthly dose was 6.5 (4.5–8.6), 4.0 (3.2–4.9), and 2.2 (1.3–3.0) µg/kg at first observation and 6.5 (4.5–8.6), 2.9 (2.1–4.0), and 1.5 (0.9–2.4) µg/kg at last observation, respectively.

The respective age-related median doses at last observation normalized to body surface area were 114 (69–218), 121 (69–166), 87 (62–120), and 98 (59–145) µg/m^2^/month at < 2, 2 to < 5, 5 to < 12, and 12 to < 18 years in the PD cohort and 155 (100–211), 83 (59–104), and 53 (35–81) µg/m^2^/ month at 2 to < 5, 5 to < 12, and 12 to < 18 years in the HD group. The dosing differences were smaller when doses were normalized to body surface area rather than body weight; however, there were only 2 patients in the age group 2 to < 5 in the HD cohort and there are only data for one observation available.

 Weight-related doses were inversely correlated with age (at last observation: r = –0.41, *p* < 0.0001 for PD, r = –0.62, *p* < 0.0001) for HD (Fig. 3A). The correlations of age and body surface area-related doses were also significant but at a lower level: r = –0.19, *p* < 0.05 for PD, r = –0.48, *p* < 0.0005) for HD (Fig. 3B). The C.E.R.A. dose normalized to body surface area was inversely correlated with Hb levels at last observation, both in the PD (r = –0.23, *p* = 0.001) and in the HD cohort (r = –0.38, *p* = 0.005) (Fig. 3C). Neither serum ferritin levels nor transferrin saturation were correlated with the C.E.R.A. dose normalized to body surface area (Fig. 3D and E).

### Safety assessment

The safety of C.E.R.A. was assessed by aggregate analysis of the hospitalization events and deaths during exposure to the drug. Hospitalizations and deaths were monitored from 6 months before the first observation under C.E.R.A. until the last observation under C.E.R.A. or, if the data were available, for 6 months after the last observation with reported C.E.R.A. exposure. This time window was chosen to ensure that no safety events were missed under potential drug exposure, since the precise dates of start and discontinuation of C.E.R.A. were not recorded. The resulting median (IQR) hospitalization surveillance time per patient was 13.5 (10.2–21.2) months in the PD and 18.3 (6.2–26.6) months in the HD cohort.

One hundred twenty-one (68%) patients on PD had at least one hospitalization while using C.E.R.A., and a total of 370 hospitalizations occurred during 270 observation years (calculated as mean observation time × number of patients), i.e. 1.37 per year of observation (Table 4). Seventy-seven percent of hospitalizations (1.03 per year) were non-elective. Of these 277 non-elective hospitalizations, the most frequent causes were infections, PD technique complications, and cardiovascular and fluid/ electrolyte imbalances: infections (56% PD-related) accounted for 85 (31%), PD technique complications for 37 (14%); and cardiovascular and electrolyte/fluid imbalance for 62 (23%) of the non-elective hospitalizations. The latter included 18 admissions of 14 patients for hypertensive crises. A total of 6 hospitalizations in 3 patients were related to anemia, including the need for 2 transfusions performed in one patient and two episodes of bleeding in another patient. No episodes of thrombotic or thromboembolic events were reported in any of the children undergoing PD.

**Table 4:** Leading causes of hospitalizations

	PD		HD	
	Patients	Hospitalizations	Patients	Hospitalizations
	*n* = 177	*n* = 370	*n* = 52	*n* = 132
Number of hospitalizations per patient, n (%)				
0	56 (32%)		16 (31%)	
1	50 (28%)		9 (17%)	
2	23 (13%)		9 (17%)	
3	15 (9%)		7 (14%)	
4	9 (5%)		1 (2%)	
5	8 (5%)		4 (8%)	
6	4 (2%)		2 (4%)	
> 6	12 (7%)		4 (8%)	
Non-elective hospitalizations, n (%)	102 (58%)	277 (77%)	32 (62%)	106 (80%)
Elective hospitalizations, n (%)	50 (28%)	84 (23%)	18 (35%)	26 (18%)
Causes of hospitalizations, n (%)				
Dialysis technique	41 (23%)	61 (17%)*	20 (39%)	43 (33%)
Infection	56 (33%)	85 (23%)	14 (27%)	30 (23%)
Cardiovascular	19 (11%)	30 (8%)	8 (15%)	12 (9%)
Gastrointestinal	19 (11%)	28 (8%)	4 (8%)	6 (5%)
Electrolyte/fluid imbalance	18 (10%)	32 (9%)	1 (2%)	4 (3%)
Neurological complication	3 (6%)	10 (3%)	2 (4%)	2 (2%)
Bone disease	6 (3%)	9 (2%)	3 (6%)	3 (2%)
Anemia	3 (2%)	6 (2%)	0 (0%)	0 (0%)
Surgery	24 (14%)	37 (10%)	9 (17%)	14 (11%)
Workup for transplantation	4 (2%)	4 (1%)	8 (15%)	9 (7%)
Annual workup	6 (3%)	6 (2%)	1 (2%)	1 (1%)
Non-compliance	2 (1%)	5 (1%)	1 (2%)	1 (1%)
Patient/family burden	1 (1%)	1 (0%)	0 (0%)	0 (0%)
Other social	2 (1%)	5 (1%)	0 (0%)	0 (0%)
Other	16 (9%)	42 (11%)	5 (10%)	7 (5%)

Percentages in patient columns represent number of patients with at least one hospitalization / number of patients per category; percentages in hospitalizations columns reflect number of hospitalizations / total number of hospitalizations over all patients per category

*HD* hemodialysis, *PD* peritoneal dialysis

*37 out of the 61 dialysis technique-related hospitalizations were non-elective

In the HD cohort, 36 patients (69%) had at least 1 hospitalization (total of 132 hospitalizations) during 83.6 observation years while using C.E.R.A., corresponding to a rate of 1.58 hospitalization events per year of observation.

One hundred six out of 132 (80%) of these hospitalizations (1.21 per year) were non-elective. Forty-three (41%) of the non-elective hospitalization events were due to HD technique complications, 30 (28%) to infections, and 16 of the 106 events (15%) were due to cardiovascular and fluid/ electrolyte imbalance events. Ten admissions in 7 patients were due to hypertensive crises. No thrombotic or thromboembolic events unrelated to dialysis access, and no blood transfusions, were reported.

Five children in the PD cohort and 2 of the children undergoing HD died while receiving C.E.R.A. (i.e., 3% of the total population), corresponding to an overall mortality rate of 19.8 cases per 1000 observation years. The causes of death included infections (*n* = 2), intracranial bleeding (*n* = 2), congestive heart failure (*n* = 2), and one case of sudden death at home.

## Discussion

In this observational study of 229 children on long-term dialysis, we provide important information on the efficacy, dosing requirements, and safety of C.E.R.A. in pediatric patients. The sources of the data analyzed here, the IPDN registries, are globally active, voluntary patient registries, in which data are collected prospectively, at baseline, and every 6 months. Since the contributing centers usually enter information from all their patients on long-term dialysis, selection bias due to preferential enrollment of patients with unique or optimal characteristics is unlikely. The electronic case report forms for the registries require complete recording of all data items, thereby excluding any potential bias from incomplete reporting. Observations were performed as part of routine clinical practice. For the analysis presented in this report, all patients with documented C.E.R.A. were analyzed; no exclusion criteria other than age less than 18 years were applied. Hence, the results of the study can be truly considered “real-world evidence”.

Previous clinical trials of C.E.R.A. in children have achieved mean Hb levels of 11.1 g/dL and 11.0 g/dL, both within the recommended 10–12 g/dL range [10, 12]. In this study, the efficacy of anemia correction with C.E.R.A. was assessed by the distribution of Hb concentrations over time of exposure. Overall, mean Hb concentrations were maintained within the 10–12 g/dL range in approximately 50% of patients in both the PD and the HD cohort. The mean Hb levels achieved and the variation of steady state Hb levels at last observation were similar to those of unmatched comparator groups from the IPDN registry (unpublished data; analysis not included in this study), indicating equally good overall anemia control with C.E.R.A. as with conventional ESAs.

Only two transfusion events were recorded, both performed in a single patient. Whereas similar proportions of children on PD displayed sub- and supratherapeutic Hb concentrations (28.2% and 24.9%, respectively), a larger proportion of the patients on HD had Hb levels below 10 g/dL (38.5%). We speculate that HD-related blood losses and more conservative, risk-averse C.E.R.A. dosing may have contributed to the lower average Hb values in this cohort. Indeed, the median monthly C.E.R.A. dose at last observation was 37% lower in the HD compared with the PD cohort. This difference was not explained by the higher fraction of younger children in the PD population, since it was also present in the 12 to < 18-year age group (PD: 3.0 versus HD 1.5 µg/kg).

The observed differences in Hb concentrations between the cohorts are also not explained by differences in the preferred route of administration. While all but 3 patients in the HD cohort received C.E.R.A. intravenously, 62% of children in the PD cohort received the drug via the subcutaneous route. Within the PD group, similar C.E.R.A. doses were used and similar Hb concentrations achieved with subcutaneous and intravenous administration. The surprisingly high rate of intravenous C.E.R.A. use in the PD group can probably be attributed to practical considerations; since C.E.R.A. is usually administered at outpatient visits, which include blood sampling, the venous access can readily be used to inject the drug to avoid the discomfort of a subcutaneous injection.

The efficacy of anemia correction was independent of age, with similar mean Hb concentration levels achieved across all age groups in both the PD and the HD cohorts. However, there was a linear inverse relationship of the body size-adjusted monthly C.E.R.A. doses with age. In both cohorts, young infants received a threefold-higher, weight-related dose than adolescents. The decreasing dosing requirement with age was less marked, albeit still significant, when the administered doses were normalized to body surface area. In addition, previous studies in pediatric populations have reported higher weight-adjusted dose requirements for ESAs in younger children with CKD [15–17]; this was also observed for C.E.R.A. in the SKIPPER study. The presence of erythropoietin receptors on nonhematopoietic cells in infants may be a reason for this relationship, as there is an increased clearance of exogenous erythropoietin that leads to less hormones to mediate erythropoietic effects [17]. Similar age-dosing associations have previously been reported for other ESAs in the pediatric PD population by the IPPN registry [17]. It appears that standardization of ESA dosing to body surface area is superior to dosing by body weight in predicting the pharmacodynamic response across the pediatric age range. Additionally, the C.E.R.A. dose normalized to body surface area was inversely correlated with Hb levels at last observation, an association that has been seen in real-world data of patients under maintenance therapy [17]. In contrast to clinical trials, in clinical practice, all pediatric patients are started on a similar dose, per recommendation or extrapolated from adult dose for non-approved drugs, and subsequent doses are changed according to the response; doses tend to increase in poor responders and decrease in very good responders, resulting in a weak inverse dose–effect relationship [17]. According to our analysis, 100 µg/m^2^ may be an appropriate monthly dose in children of all ages to achieve or maintain Hb levels in the target range.

The observed lack of correlation of C.E.R.A. doses with ferritin levels and transferrin saturation in patients on both PD and HD indicates that the variability of ESA dose requirements was not explained by iron status or the presence of an inflammatory state.

While comparisons between trials and real-world data should be interpreted with caution, this real-world evidence supports existing evidence from clinical trials of C.E.R.A. in pediatric patients. A clinical trial (DOLPHIN) including pediatric patients on HD and maintenance IV ESA treatment established a conversion factor (4 mg every 4 weeks for each weekly dose of 125 IU epoetin alfa/beta or 0.55 μg darbepoetin) to calculate the starting dose for effectively switching pediatric patients from previous ESAs to C.E.R.A., which was further supported by SKIPPER: a clinical trial including dialyzed and non-dialyzed CKD patients on maintenance subcutaneous ESA treatment [10, 12].

Our study did not yield any major safety signals. Neither the rate nor the distribution of causes of non-elective hospitalizations differed from those expected for pediatric dialysis populations. Infections, the majority of which were related to the dialysis procedure, were the leading cause of hospitalizations in children on PD, whereas technical complications (dialysis access dysfunction) were the most common cause of hospitalizations in patients on HD. Catheter clotting and fistula thrombosis accounted for a significant fraction of catheter dysfunction; the incidence of these events in the 52 children exposed to C.E.R.A. was similar to that previously reported for the entire IPHN cohort [18]. Seven percent (18/277) and 9% (10/106) of the non-elective hospitalization events in children on PD and HD, respectively, were due to hypertensive crises; these figures are well within the expected incidence for a pediatric dialysis population. No thrombotic or thromboembolic events unrelated to dialysis access, and no blood transfusions, were reported.

As expected in a pediatric dialysis population, mortality was low, with 7 fatal outcomes, corresponding to 2 cases per 100 patient years. This mortality rate is in keeping with the figures recently reported for the entire IPPN cohort [19].

We acknowledge several limitations of this registry study. In the present study, centers from 11 countries in West and East Europe, Southeast and East Asia, and North and South America were represented. While this worldwide representation should minimize selection bias, the fact that participation in IPPN and IPHN is voluntary, leaves the possibility of differences in treatment practices between contributing centers and centers not contributing to the registries. In the registries, follow-up data are obtained once every 6 months and treatment information is available only as 6-month snapshots without an actual starting date. Hence, the precise exposure times to C.E.R.A. at the time of Hb measurement are not known. The median duration of exposure was 6.1 months, and the majority of patients had a C.E.R.A. duration of exposure of 0–12 months. For patients on PD and HD, 49% and 39% of patients, respectively, had a C.E.R.A. exposure of less than or equal to 6 months, which means only one observation was recorded in the registry. To address this limitation, a separate analysis was conducted for Hb concentration and C.E.R.A. dose for a subset of patients with two or more observations captured in the registry, which showed similar results to the entire cohort. However, the analysis and interpretation of Hb stability is limited as we only have the 6-monthly snapshots. Also, hospitalizations are reported every 6 months retrospectively for the preceding 6 months without an actual date. For this reason, it is not known for certain that hospitalizations occurred while patients were exposed to C.E.R.A. when the drug was started or discontinued between two consecutive observations; hence, the number of hospitalizations under C.E.R.A. exposure may have been slightly overestimated. In addition, there was no information recorded by the registries regarding the possible causes of hyperresponsiveness to C.E.R.A. in patients on PD and HD. Also, no control group of children was exposed to ESAs other than C.E.R.A. The IPPN and IPHN registries will continue to collect efficacy and safety data, including data from patients on C.E.R.A. and other ESAs; additionally, further safety items are to be included in the case report forms.

In summary, our real-world evidence study of children on long-term dialysis suggests that C.E.R.A. may allow for efficient management of anemia in this population. Body weight-related dosing requirements decrease with age. C.E.R.A. also appears to have a favorable safety profile; the analysis of hospitalization rate and causes as well as patient mortality did not reveal any safety signals.

### Supplementary Information

Below is the link to the electronic supplementary material.

Graphical Abstract (PPTX 129 KB)

Supplementary file2 (DOCX 59 KB)

## Data Availability

The datasets analyzed during this study are not publicly available because they are the proprietary property of the IPDN network: http://www.pedpd.org/
