# Supplementary material for: Real-world evidence on the dosing and safety of C.E.R.A. in pediatric dialysis patients: findings from the International Pediatric Dialysis Network registries
Source: Pediatr Nephrol. 2023 Aug 11;39(3):807–18. doi: 10.1007/s00467-023-05977-z (PMC10817843; doi:10.1007/s00467-023-05977-z)
Supplement: Supplementary file 1 — Graphical Abstract (PPTX 129 KB) [file 467_2023_5977_MOESM1_ESM.pptx]

## Slide 1
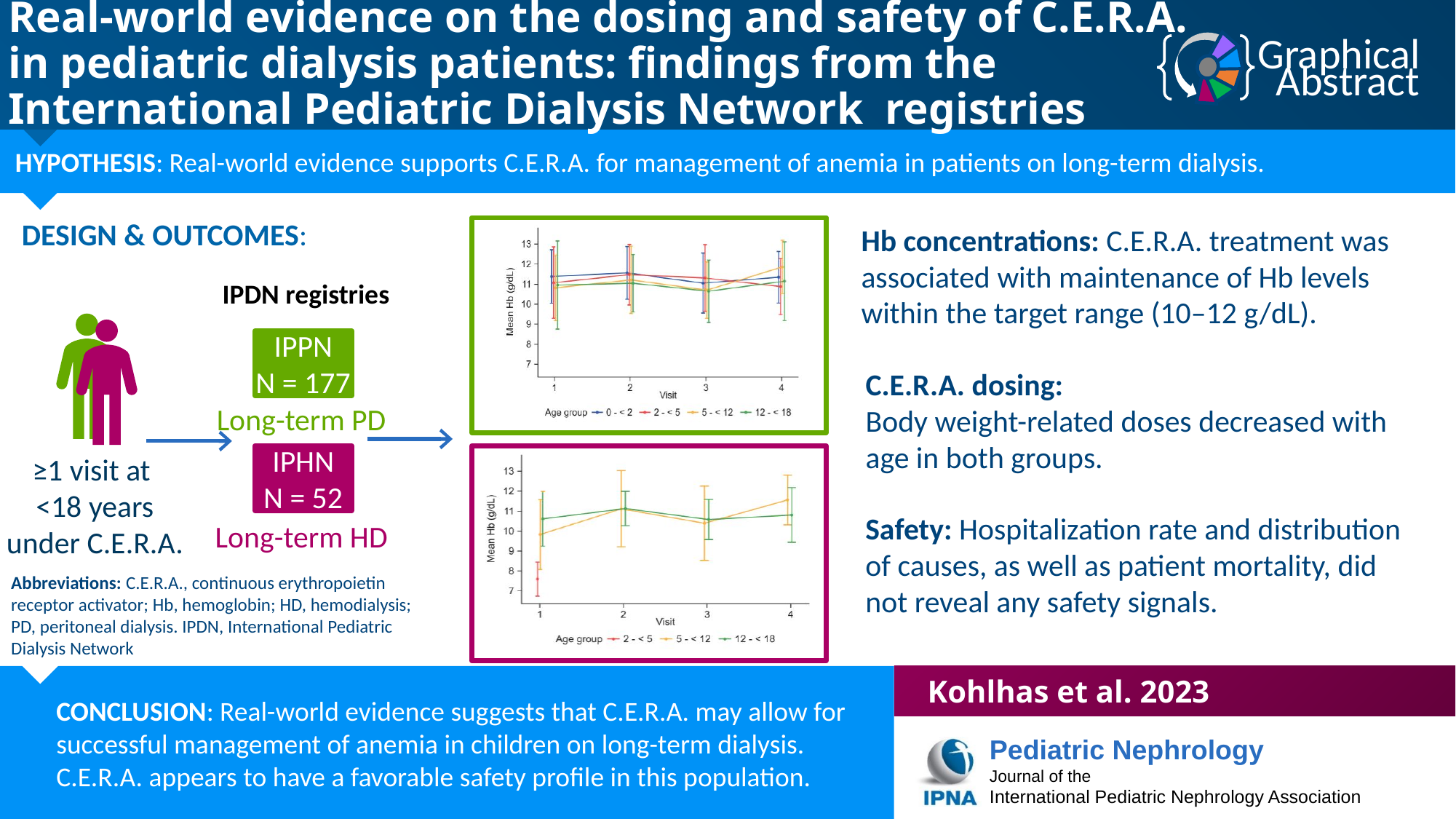

Real-world evidence on the dosing and safety of C.E.R.A. in pediatric dialysis patients: findings from the International Pediatric Dialysis Network registries
HYPOTHESIS: Real-world evidence supports C.E.R.A. for management of anemia in patients on long-term dialysis.
DESIGN & OUTCOMES:
Hb concentrations: C.E.R.A. treatment was associated with maintenance of Hb levels within the target range (10–12 g/dL).
IPDN registries
IPPN
N = 177
IPHN
N = 52
C.E.R.A. dosing:
Body weight-related doses decreased with
age in both groups.
Long-term PD
≥1 visit at
<18 years under C.E.R.A.
Safety: Hospitalization rate and distribution of causes, as well as patient mortality, did not reveal any safety signals.
Long-term HD
Abbreviations: C.E.R.A., continuous erythropoietin receptor activator; Hb, hemoglobin; HD, hemodialysis;
PD, peritoneal dialysis. IPDN, International Pediatric Dialysis Network
Kohlhas et al. 2023
CONCLUSION: Real-world evidence suggests that C.E.R.A. may allow for successful management of anemia in children on long-term dialysis.
C.E.R.A. appears to have a favorable safety profile in this population.
