# Supplementary material for: Real-world evidence on the dosing and safety of C.E.R.A. in pediatric dialysis patients: findings from the International Pediatric Dialysis Network registries
Source: Pediatr Nephrol. 2023 Aug 11;39(3):807–18. doi: 10.1007/s00467-023-05977-z (PMC10817843; doi:10.1007/s00467-023-05977-z)
Supplement: Supplementary file 2 — Supplementary file2 (DOCX 59 KB) [file 467_2023_5977_MOESM2_ESM.docx]

**Real-world evidence on the dosing and safety of C.E.R.A. in pediatric dialysis patients: findings from the International Pediatric Dialysis Network (IPDN) registries**

Laura Kohlhas^1^ · Milena Studer^2^ · Loes Rutten-Jacobs^2^ · Sylvie Meyer Reigner^2^ · Anja Sander^1^ ·
Hui-Kim Yap^3^ · Karel Vondrak^4^ · Paula A. Coccia^5^ · Francisco Cano^6^ · Claus Peter Schmitt ^7^ ·
Bradley A. Warady^8^ · Franz Schaefer^7^

^1^Institute of Medical Biometry, University of Heidelberg, Heidelberg, Germany

^2^F. Hoffmann-La Roche Ltd, Basel, Switzerland (ORCID iD: 000-003-3223-885X)

^3^Department of Paediatrics, Yong Loo Lin School of Medicine, National University of Singapore, Singapore

^4^Department of Pediatrics and Transplantation Center, University Hospital Motol, 2nd Medical Faculty Prague, Charles University Prague, Prague, Czech Republic

^5^Division of Pediatric Nephrology, Hospital Italiano de Buenos Aires, Buenos Aires, Argentina

^6^Division of Pediatric Nephrology, Hospital Dr. Luis Calvo Mackenna, Facultad de Medicina, Universidad de Chile, Chile (ORCID iD: 0000-0003-4425-7193)

^7^Division of Pediatric Nephrology, Center for Pediatrics and Adolescent Medicine, Heidelberg, Germany

^8^Division of Pediatric Nephrology, Children’s Mercy Kansas City, Kansas City, MO, USA

**Corresponding author**

Prof. Dr. Franz Schaefer

Center for Pediatrics and Adolescent Medicine

Im Neuenheimer Feld 430

69120 Heidelberg, Germany

E-Mail: [Franz.Schaefer@med.uni-heidelberg.de](mailto:Franz.Schaefer@med.uni-heidelberg.de)

Telephone Number: +49 6221 563 2396

**Supplementary Table 1** Patient characteristics of patients with ≥2 observations at first visit.

|  | **PD** | **HD** |
| --- | --- | --- |
|  | n = 108 | n = 35 |
| Female sex, n (%) | 39 (36%) | 15 (43%) |
| Mean age, years (±SD) | 9.2 (5.5) | 13.5 (3.1) |
| Age 0 to < 2 years, n (%) | 13 (12.0%) | 0 (0%) |
| Age 2 to < 5 years, n (%) | 21 (19.4%) | 0 (0%) |
| Age 5 to < 12 years, n (%) | 35 (32.4%) | 12 (34.3%) |
| Age 12 to < 18 years, n (%) | 39 (36.1%) | 23 (65.7%) |
| Geographic region, n (%) |  |  |
| Europe* | 63 (58%) | 34 (97%) |
| Asia^†^ | 27 (25%) | 0 (0%) |
| Latin America^‡^ | 18 (17%) | 0 (0%) |
| North America^§^ | 0 (0%) | 1 (3%) |
| Primary renal diagnosis, n (%) |  |  |
| -CAKUT | 55 (51%) | 23 (66%) |
| -Other | 53 (49%) | 12 (34%) |
| Defined syndrome^¶^, n (%) | 18 (17%) | 4 (11%) |
| Mean body weight, kg (±SD) | 27.9 (16.8) | 43.6 (18.2) |
| Mean body surface area, m^2^ (±SD) | 1.0 (0.4) | 1.3 (0.4) |
| Mean systolic blood pressure, mmHg (±SD) | 112.9 (19.2) | 130.1 (15.1) |
| Mean diastolic blood pressure, mmHg (±SD) | 71.0 (15.2) | 77.8 (13.3) |
| Median time on dialysis, months (IQR) | 8.0 (1.9–25.2) | 2.0 (1.2–14.3) |
| C.E.R.A. route of administration, n (%) |  |  |
| - Intravenous | 32 (40%) | 33 (94%) |
| - Subcutaneous | 48 (60%) | 2 (6%) |
| - Missing | 28 | 0 |
| Prior ESA, n (%) |  |  |
| - Alfa erythropoietin | 10 (16%) | 1 (17%) |
| - Beta erythropoietin | 35 (57%) | 0 (0%) |
| - Darbepoetin | 8 (13%) | 4 (67%) |
| - Delta erythropoietin | 8 (13%) | 0 (0%) |
| - No | 0 | 1 (17%) |
| - Missing | 47 | 29 |
| ACE inhibitor/ARB, n (%) |  |  |
| - No | 71 (70%) | 26 (74%) |
| - Yes | 31 (30%) | 9 (26%) |
| - Missing | 6 | 0 |
| Iron therapy, n (%) |  |  |
| - No | 24 (22%) | 3 (9%) |
| - Yes | 84 (78%) | 32 (91%) |
| Median serum ferritin, ng/ml (IQR) | 183 (82–308) | 267 (138–367) |
| Median transferrin saturation, % (IQR) | 31.8 (19.5–38.0) | 31.0 (19.0–35.7) |

Data are given as n (%), mean (SD), or median (interquartile range) as appropriate. *ACE* angiotensin-converting-enzyme, *ARB* angiotensin II receptor blocker, *CAKUT* congenital anomalies of the kidney and urinary tract, *C.E.R.A.* continuous erythropoietin receptor activator, *ESA* erythropoiesis-stimulating agent*, HD* hemodialysis, *IQR* interquartile range, *PD* peritoneal dialysis, *SD* standard deviation.

* Belgium, Czech Republic, France, and Germany.

^†^ South Korea, Malaysia, and Singapore.

^‡^ Argentina, Chile, and Colombia.

^§^ The United States.

^¶^ Defined syndromes are disorders that also affect organs other than the kidney, such as Bardet-Biedl syndrome, VACTERL syndrome, or even Down syndrome. Patients with defined syndromes often have additional comorbidities that may modify outcomes. They are less relevant with respect to anemia treatment.

**Supplementary Table 2** C.E.RA. duration of exposure of patients with ≥2 observations.

|  | **PD** | **HD** |
| --- | --- | --- |
|  | n = 108 | n = 35 |
| Median duration of exposure, months (IQR) | 11.8 (6.5–18.9) | 14.8 (11.7–30.1) |
| C.E.R.A. duration of exposure, n (%) |  |  |
| - ≤6 months | 17 (15.7%) | 3 (8.6%) |
| - 6–12 months | 38 (35.2%) | 6 (17.1%) |
| - 12–18 months | 22 (20.4%) | 13 (37.1%) |
| - 18–24 months | 12 (11.1%) | 3 (8.6%) |
| - 24–36 months | 9 (8.3%) | 8 (22.9%) |
| - 36–48 months | 5 (4.6%) | 1 (2.9%) |
| - 48–60 months | 2 (1.9%) | 1 (2.9%) |
| - 60–71 months | 2 (1.9%) | 0 (0%) |
| - 84–96 months | 1 (0.9%) | 0 (0%) |

*C.E.R.A.* continuous erythropoietin receptor activator*, HD* hemodialysis, *IQR* interquartile range, *PD* peritoneal dialysis.

**Supplementary Table 3**  Hemoglobin level and C.E.R.A. dose (absolute and normalized to body weight or body surface area) at first and last observation on treatment, stratified by route of administration of patients with ≥2 observations.

|  | **PD**  **subcutaneous** | | **PD**  **intravenous** | | **PD**  **all routes** | | **HD** | |
| --- | --- | --- | --- | --- | --- | --- | --- | --- |
|  | First obs.  n = 48 | Last obs. n = 46 | First obs.  n = 32 | Last obs.  n = 39 | First obs.  n *=* 108 | Last obs.  n *=* 108 | First obs.  n = 35 | Last obs. n = 35 |
| Hemoglobin, g/dL | 11.3 (2.1) | 10.9 (1.7) | 10.9 (1.9) | 11.4 (1.4) | 11.1 (1.9) | 11.0 (1.6) | 10.4 (1.4) | 10.7 (1.5) |
| C.E.R.A. monthly dose, µg | 90 (50–155) | 100 (50–161) | 100 (50–120) | 100 (50–120) | 100 (50–120) | 100 (50–129) | 107 (80–129) | 80 (54–107) |
| C.E.R.A. monthly dose, µg/kg | 3.6 (2.7–5.1) | 3.6 (2.5–5.1) | 3.5 (2.5–5.8) | 3.0 (1.9–4.7) | 3.4 (2.3–5.3) | 3.5 (2.1–5.0) | 2.8 (1.6–3.9) | 1.8 (1.0–2.6) |
| C.E.R.A. monthly dose, µg/m^2^ | 93 (70–122) | 95 (64–141) | 95 (67–149) | 89 (54–123) | 91 (67–133) | 93 (58–139) | 89 (57–113) | 57 (37–81) |

Hemoglobin is given as mean (SD), doses as median (interquartile range). The results of three patients on HD treated subcutaneously are not shown. *C.E.R.A.* continuous erythropoietin receptor activator, *HD* hemodialysis, *Obs* observations, *PD* peritoneal dialysis, *SD* standard deviation.
